# The efficacy and safety of Trilaciclib in preventing chemotherapy-induced myelosuppression: a systematic review and meta-analysis of randomized controlled trials

**DOI:** 10.3389/fphar.2023.1157251

**Published:** 2023-05-25

**Authors:** Jingyue Qiu, Dandan Sheng, Fei Lin, Peng Jiang, Ning Shi

**Affiliations:** ^1^ Pharmaceutical Department, PLA Strategic Support Force Medical Center, Beijing, China; ^2^ Department of Pharmacy, The First Affiliated Hospital of Chengdu Medical College, Chengdu, China; ^3^ Clinical Medical College, Chengdu Medical College, Chengdu, China; ^4^ Medical Team, PLA Strategic Support Force Integrated Training Team, Beijing, China

**Keywords:** CDK4/6 inhibitor, trilaciclib, chemotherapy, myelosuppression, meta-analysis

## Abstract

**Background:** This study aims to assess the clinical efficacy and safety of Trilaciclib in preventing chemotherapy-induced myelosuppression in adult patients through meta-analysis.

**Methods:** The PubMed, Embase, Cochrane Library, Clinical Trials, EU Clinical Trials Register, and International Clinical Trials Registry Platform were searched up to 25 October 2022. Only randomized controlled trials (RCTs) comparing the clinical outcomes of Trilaciclib and Trilaciclib plus chemotherapy for treating malignant cancers in adult patients were included. The primary outcome included the incidence of SN, FN, the DSN, and administration of ESAs, G-CSFs, and RBC or platelet transfusions, while the secondary outcomes included the risk of adverse events (AEs) and severe adverse events (SAEs).

**Results:** In total, four randomized controlled trials (RCTs) involving 345 patients with SCLC or breast cancer were included in this meta-analysis. Results showed that administration of Trilaciclib significantly reduced the occurrence of SN (19.3% vs. 42.2%, OR = 0.31), FN (3.22% vs. 6.72%, OR = 0.47), anemia (20.5% vs. 38.2%, OR = 0.38) and shortened the DSN during treatment. The proportion of patients receiving therapeutic use of ESAs (4.03% vs. 11.8%, OR = 0.31), G-CSF (37.0% vs. 53.5%, OR = 0.52), RBC transfusions (19.8% vs. 29.9%, OR = 0.56) was also statistically lower in the experimental group than in the control group. Meanwhile, the ORR, overall survival, and progress-free survival of the two groups were identical, and no negative impact of Trilaciclib on the clinical outcomes of chemotherapy treatments was found. Other chemotherapy-induced adverse events (AEs) and severe adverse events (SAEs) like diarrhea, fatigue, nausea, and vomiting were identical regardless of Trilaciclib usage.

**Conclusion:** Trilaciclib demonstrated its efficacy in reducing the occurrence of chemotherapy-induced myelosuppression and utilization of supportive care interventions without undermining the clinical benefits of chemotherapy regimens during treatment with an acceptable safety profile.

## 1 Introduction

Chemotherapy is currently the cornerstone for treating many cancers like extensive-stage small cell lung cancer (SCLC), triple-negative breast cancer, etc. (Horn et al., 2018; Goldman et al., 2021; Bianchini et al., 2022). However, standard chemotherapy regimens are usually associated with myelosuppression, which may not only affect the therapeutic effect of chemotherapy but also lead to life-threatening complications like secondary infections, anemia, and bleeding. It is reported that more than 60% of patients receiving chemotherapy treatments for SCLC had at least one grade ≥3 myelosuppressive AE during treatment (Epstein et al., 2022). The incidence of chemotherapy-induced grade ≥3 neutropenia, anemia, and thrombocytopenia was 44.9%, 44.1%, and 25.4%, respectively (Epstein et al., 2022). Currently, chemotherapy-induced myelosuppression (CIM) is mainly managed with dose delay/reductions, administration of ESAs or G-CSFs, and RBC or platelet transfusions, which are burdensome to the patients and may bring other undesirable side effects (Kogan et al., 2019; Crawford et al., 2020; Epstein et al., 2020). Severe CIM affects the clinical outcome of chemotherapy treatment and imposes a financial burden on the patients and the healthcare system.

Trilaciclib is a selective and reversible inhibitor of cell cycle protein-dependent kinases 4 and 6 (CDK4/6) approved by the FDA in February 2021 as a first-in-class myeloprotective agent. Intravenous administration of Trilaciclib prior to chemotherapy can transiently arrest the CDK4/6-dependent hematopoietic stem/progenitor cells (HSPCs) and lymphocytes in the G1 phase of the cell cycle, preventing the DNA damage and apoptosis of these cells after exposure to chemotherapeutic agents (He et al., 2017). Moreover, Trilaciclib protected multilineage myeloid cells like neutrophils, red blood cells, and platelets from CIM in SCLC patients in multiple clinical trials without compromising chemotherapy efficacy and patient survival, reduced the need for supportive care interventions after treatment, improved the quality of life of the patients and provided significant clinical benefits (Dómine Gómez et al., 2021; Ferrarotto et al., 2021; Hart et al., 2021; Hussein et al., 2021). However, in another study assessing the myeloprotective effect of Trilaciclib in patients with metastatic triple-negative breast cancer, no significant differences were observed in myelosuppression endpoints between groups of Trilaciclib plus chemotherapy and chemotherapy alone, though significantly longer PFS and OS were observed (Tan et al., 2019). Moreover, some experts believed that the clinical benefits that Trilaciclib may bring to the patients should be confirmed with more extensive phase III trials and that more research was needed (Powell and Prasad, 2021). Therefore, it is necessary to systematically evaluate the preventive effect of Trilaciclib in multilineage CIM.

In this study, the CDK4/6 inhibitor Trilaciclib was investigated. Its clinical benefits and safety were compared in patients treated with therapeutic chemotherapy agents to provide a reference for clinical application.

## 2 Materials and methods

### 2.1 Study search and selection

We searched PubMed, Embase, the Cochrane Library, Clinical Trials, the EU Clinical Trials Register, and the International Clinical Trials Registry Platform (ICTRP) using “Trilaciclib” or “Cosela” or “G1T28” as search terms. ENDNOTE X 8 was used to remove the duplicate record. And after removing duplicate records from the search results, two researchers screened and reviewed each study independently. Any disagreement in the process was resolved by consulting a third researcher. All the data were extracted from the included studies, including the authorship, year of publication, study design, study duration, study site, study population, chemotherapy regimens and the comparators, clinical outcomes, and risk of AEs. The included studies should meet the following criteria: patients diagnosed with malignant cancer; age was ≥18 years old; intervention of chemotherapy, and comparison of chemotherapy vs. chemotherapy plus Trilaciclib; RCT; reporting of the efficacy outcome, including the incidence of CIM, the utilization of supportive care interventions; and the safety outcome. In this study, no ethical approval was necessary for meta-analysis in our institute.

### 2.2 Outcome measurement

The study’s primary outcome was the rate of CIM-related AEs and the utilization of supportive care interventions. We systemically analyzed the rate of severe neutropenia (SN), febrile neutropenia (FN), the administration of erythropoiesis-stimulating agents (ESA), granulocyte colony-stimulating factors (G-CSFs), RBC or platelet transfusions, and the duration of severe neutropenia (DSN) to evaluate the protective effect of Trilaciclib from CIM. AEs like anemia, diarrhea, fatigue, leukopenia, nausea, neutropenia, thrombocytopenia, and vomiting were also statistically analyzed to evaluate the potential safety of Trilaciclib. The impact of Trilaciclib on the overall response rate (ORR), overall survival (OS), and progress-free survival (PFS) was analyzed to determine the comprehensive effect on the patients.

### 2.3 Data analysis

The included studies’ quality and associated risk of bias were performed using the Cochrane risk-of-bias tool (Higgins et al., 2011). Two researchers subjectively reviewed all included studies and rated them “low risk,” “high risk,” or “unclear risk” according to the judgment items in the tool. All statistical analyses were performed by using Review Manager version 5.3. Pooled odds ratios (ORs) and 95% confidence intervals (CIs) were used to measure the association between outcomes and the use of Trilaciclib. Study heterogeneity was presented using the Chi-squared-based Cochran’s Q statistic and I^2^. The heterogeneity was considered significant when the *p* < 0.10 or I^2^ > 50%. The fixed-effect model was used when data were homogenous, and the random-effect model was used when data were significantly heterogeneous. A sensitivity analysis was conducted using a leave-one-out approach.

## 3 Results

### 3.1 Search and study characteristics

**FIGURE 1:**
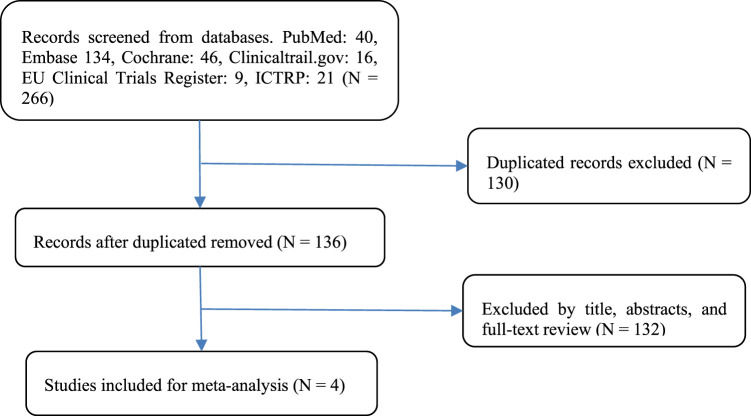
Flowchart of the study selection process.

**FIGURE 2:**
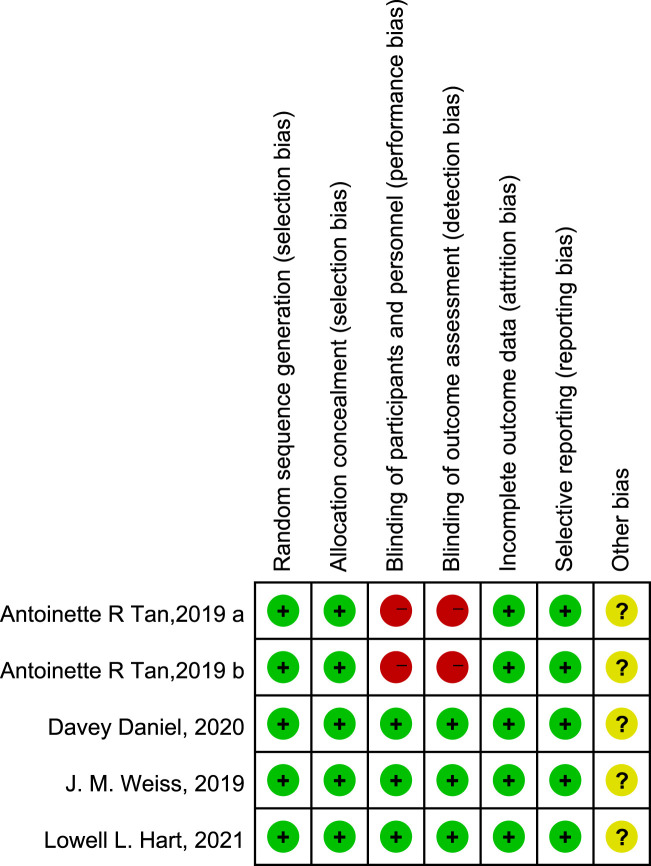
Risk of bias summary.

A flow diagram of the study selection is presented in Figure 1. The search program yielded 266 references from PubMed (N = 40), Embase (N = 134), Cochrane Library (N = 46), Clinical Trials (N = 16), EU Clinical Trials Register (N = 9), ICTRP (N = 21). After excluding 130 duplicates, the remaining 136 articles were screened. Four multicenter, intention-to-treat RCTs published between 2019 and 2021 met the inclusion criteria and were included in the systematic review and meta-analysis. Three of the four studies were double-blind, and one was open-label (Table 1). All four studies were conducted in multiple countries. Among the 347 participants enrolled, 193 patients received Trilaciclib plus chemotherapy (experimental group), 154 patients received chemotherapy alone (control group), 169 patients were male, and 178 patients were female (Table 2). Weiss’s study consists of part 1 (open-label, dose-finding) and part 2 (RCT, double-blind, placebo-controlled); only part 2 patients were included. In Weiss’s study, two patients were excluded from data analysis for violation of study procedures, so the number of patients included for analysis in the experimental and control groups was 192 and 153, respectively. In Tan’s study, two subgroups with different schedules of Trilaciclib administration (on days 1, 8, and 2, 9, respectively) were designed and analyzed independently. Trilaciclib was administered to patients at the recommended dose of 240 mg/m^2^ 0–3 days before chemotherapy started. Dose modifications were allowed for chemotherapy but not for Trilaciclib. All patients were diagnosed with SCLC or breast cancer. In the control group, the four studies used gemcitabine/carboplatin (G/P) therapy, etoposide/carboplatin/atezolizumab (E/P/A) therapy, etoposide/carboplatin (E/P) therapy and topotecan, respectively. Three studies focused on SCLC, with the other targeting metastatic triple-negative breast cancer. Prophylactic administration of ESAs or G-CSF was prohibited in cycle 1 to avoid interference with the results, but therapeutic ESAs or G-CSF usage was allowed in all cycles. The risk of bias in the included studies is presented in Figure 2, 3. Tan’s study was found to have a high risk of bias in the domains of blinding of participants and performance and blinding of outcome assessment. All trials were designed and conducted in accordance with the Declaration of Helsinki and International Council for Harmonization Good Clinical Practice guidelines.

**TABLE 1:** Characteristics of selected studies.

Study, year published	Intervention		Patient number		Study duration	Study population
	Control	Experimental	Control	Experimental		
J. M. Weiss, 2019	E/P plus placebo	E/P plus Trilaciclib	37	38	between June 2015 and February 2019	≥18 years, histologically or cytologically confirmed ES-SCLC.
Davey Daniel, 2020	Placebo prior to E/P/A	Trilaciclib prior to E/P/A	53	54	between June 2017 and February 2018	≥18 years, with confirmed ES-SCLC.
Lowell L. Hart, 2021	Placebo prior to topotecan	Trilaciclib prior to topotecan	29	32	between October 2015 and October 2021	≥18 years, with confirmed diagnosis of ES-SCLC.
Antoinette R Tan,2019	G/P plus placebo	G/P plus Trilaciclib (D1+D8)	34	33	between February 2017 and May 2018	≥18 years, recurrent or metastatic triple-negative breast cancer who had no more than two previous lines of chemotherapy
		G/P plus Trilaciclib (D2+D9)		35		

**TABLE 2:** characteristics of enrolled patients.

Study, year published	Group	Patient number	Baseline						
			Sex		Region		Age/years		
			Female	Male	USA	EX-USA	Median	18 to <65	≥65
J. M. Weiss, 2019	Control	37	11	27	39	38	66	17	21
	Experimental	38	12	27			64	20	19
Davey Daniel, 2020	Control	53	19	34	20	34	64 (46–83)	27	27
	Experimental	54	13	41	22	31	65 (45–81)	27	26
Lowell L. Hart, 2021	Control	29	12	17	18	11	64 (47–82)	18	11
	Experimental	32	10	22	14	18	62 (47–77)	20	12
Antoinette R Tan, 2019	Control	34	34	0	28	6	55 (43–64)	26	8
	Experimental	33	32	1	28	5	55 (47–66)	24	9
	Experimental	35	35	0	27	8	55 (49–65)	26	9

**FIGURE 3:**
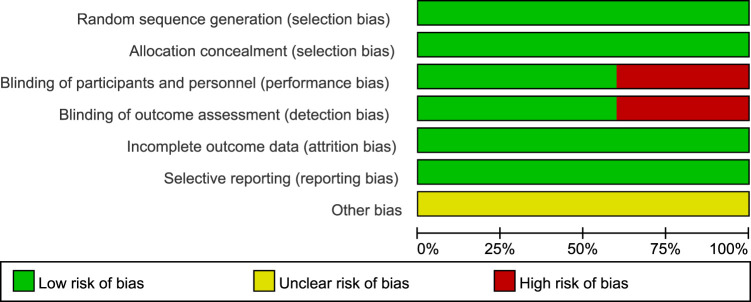
Risk of bias graph.

### 3.2 Clinical response

**FIGURE 4:**
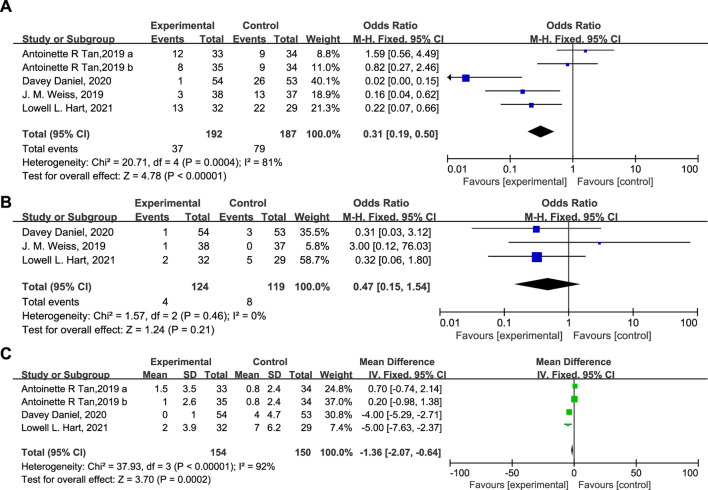
The statistical difference of SN **(A)**, FN **(B)**, and DSN **(C)** in the experimental and control group.

According to the results, 37 patients (19.3%) in the experimental group and 79 patients (42.2%) in the control group experienced SN (Figure 4A, OR = 0.31, 95% CI = 0.19–0.50, I^2^ = 81%), and 4 (3.22%) and 8 (6.72%) patients in the experimental and control group experienced FN respectively (Figure 4B, OR = 0.47, 95% CI = 0.15–1.54, I^2^ = 0%). Moreover, the DSN in the experimental group is significantly shorter than in the control group (Figure 4C, Mean Difference −1.36 days, 95% CI = −2.07−0.64, I^2^ = 92%), implying that administration of Trilaciclib prior to chemotherapy efficiently reduced the CIM-related SN and FN and shortened the DSN during treatment.

**FIGURE 5:**
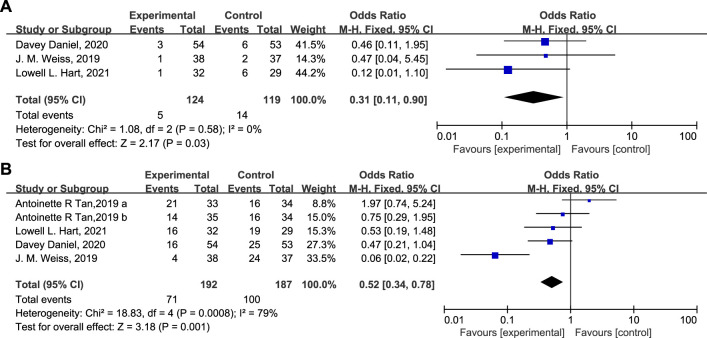
Therapeutic use of ESA **(A)** or G-CSF **(B)** in the experimental and control group.

ESA was administered to 5 patients (4.03%) in the experimental group versus 14 patients (11.8%) in the control group (Figure 5A, OR = 0.31, 95% CI = 0.11–0.90, I^2^ = 0%). The percentage of patients receiving G-CSF in the experimental and control groups was 37.0% and 53.5%, respectively (Figure 5B, OR = 0.52, 95% CI = 0.34–0.78, I^2^ = 79%). The proportion of patients receiving therapeutic use of ESAs and G-CSF was statistically lower in the experimental group than in the control group.

**FIGURE 6:**
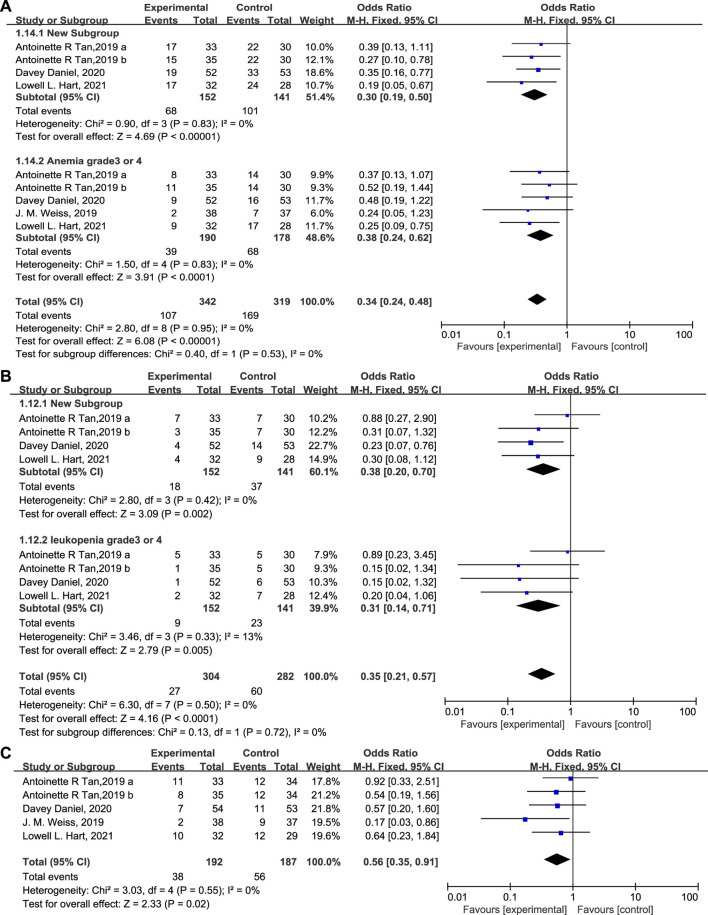
The occurrence of anemia **(A)** and leukopenia **(B)** and the proportion of patients with RBC **(C)** transfusions in the experimental and control group.

**FIGURE 7:**
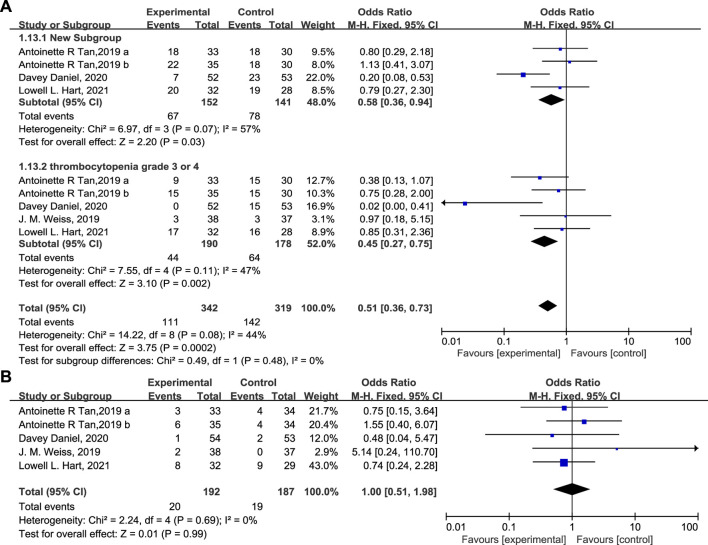
The occurrence of thrombocytopenia **(A)** and the proportion of patients with platelet transfusions **(B)** in the experimental and control group.

The percentage of patients with grade 3/4 anemia (Figure 6A, 20.5% vs. 38.2%, OR = 0.38, 95% CI = 0.24–0.62, I^2^ = 0) and leukopenia (Figure 6B, OR = 0.31, 95% CI = 0.14–0.71, I^2^ = 13%) was significantly lower in the experimental group than in the control group, in accordance with the proportion of patients receiving RBC transfusions (Figure 6C, 19.8% vs. 29.9%, OR = 0.56, 95% CI = 0.35–0.91, I^2^ = 0) on/after week 5. There are also fewer patients experiencing grade 3/4 thrombocytopenia (Figure 7A, OR = 0.45, 95% CI = 0.27–0.75, I^2^ = 47%) in the experimental group than in the control group. While the proportion of patients with platelet transfusions was identical in both groups (Figure 7B, 10.4% vs. 10.2%, OR = 1.00). It could thus be concluded that Trilaciclib reduced the occurrence of severe anemia, leukopenia, and thrombocytopenia and the need for RBC transfusions but had no impact on platelet transfusions.

**FIGURE 8:**
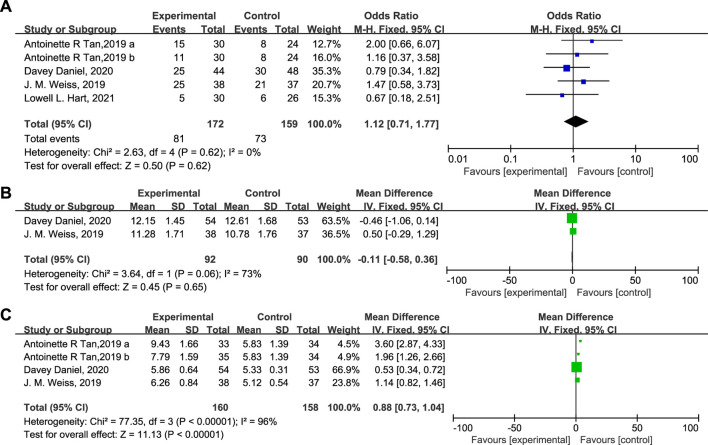
The impact of Trilaciclib on the ORR **(A)**, OS **(B)**, and PFS **(C)** in the experimental and control group.

The influence of Trilaciclib on the ORR, OS, and PFS is shown in Figure 8. As can be seen, the ORR (OR = 1.12, 95% CI = 0.71–1.77, I^2^ = 0%), OS (Mean Difference −0.11, 95% CI = −0.58 – 0.36, I^2^ = 73%), and PFS (Mean Difference 0.88, 95% CI = 0.73–1.04, I^2^ = 96%) of the two groups were identical. Moreover, fewer patients experienced chemotherapy dose delays/reductions in the Trilaciclib arm than in the placebo arm, which helps to ensure the delivery of complete cycles of chemotherapy regimens. Administration of Trilaciclib showed no negative impact on the antitumor activity of chemotherapy treatments.

**FIGURE 9:**
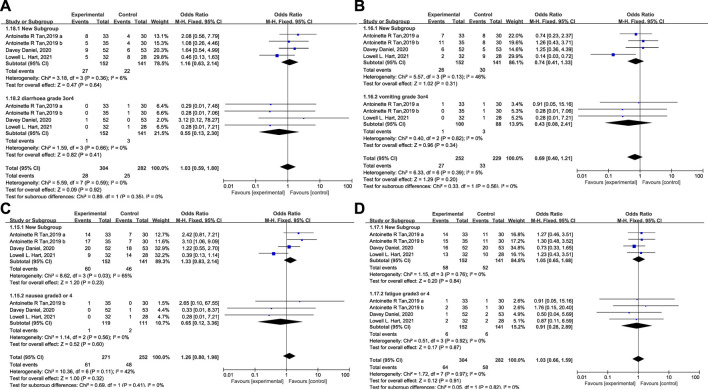
The occurrence of diarrhea **(A)**, vomiting **(B)**, nausea **(C)**, and fatigue **(D)** in the experimental and control group.

A statistical analysis of other drug-related AEs like vomiting, nausea, diarrhea, and fatigue is presented in Figure 9. No clinically relevant increase in toxicity was reported. The incidence of these AEs in both groups was identical, and grade 3/4 of these events were rare. No Trilaciclib-related grade 3/4 SAEs occurred, demonstrating that Trilaciclib has an acceptable safety profile.

## 4 Discussion

Neutropenia and anemia are the most common side effects of CIM that are detrimental to chemotherapy treatments and are increasingly recognized as an important clinical issue that needs to be more efficiently managed. Trilaciclib was the first drug approved by the FDA to prevent CIM. By transiently arresting CDK4/6-dependent cells (like HSPCs and lymphocytes) in the G1 phase of the cell cycle, Trilaciclib protected these cells from cytotoxic chemotherapy and favorably altered the tumor immune microenvironment (Lai et al., 2020). Moreover, Trilaciclib has been shown to increase tumor cells’ sensitivity to immune checkpoint inhibitors and prolong the duration of the antitumor responses in preclinical models (Deng et al., 2018; Lai et al., 2020). This supports the clinical trial of combining Trilaciclib with chemotherapy in patients with cancer. As SCLC tumor cells replicate independently of the CDK4/6 pathway, it is reasonable to conclude that Trilaciclib would achieve its efficacy without undermining the cytotoxic effect of chemotherapy agents on tumor cells, as has been demonstrated in multiple preclinical and clinical trials (Roberts et al., 2020).

In this meta-analysis of data from four phase 2 RCTs in patients with ES-SCLC and metastatic triple-negative breast cancer, administration of Trilaciclib prior to chemotherapy significantly reduced the occurrence of SN and FN and shortened the DSN during treatment. The use of supportive-care interventions like the administration of ESAs, G-CSF, and RBC transfusions on/after week 5 was also statistically reduced. Given the restricted use of ESAs and limited blood supplies in the context of COVID-19, this is especially helpful in relieving patients and the healthcare system from CIM-related anemia (Bohlius et al., 2019). Meanwhile, both groups’ OS, PFS, and ORR were identical, implying that Trilaciclib protected patients from CIM without compromising the clinical benefits of chemotherapy treatments or bringing other unexpected side effects. The median age of the included patients was >55 years old. Considering that elderly patients were more frequently associated with CIM, the clinical benefit of Trilaciclib was more convincing. Trilaciclib showed its potential as a new standard of supportive care for patients receiving myelosuppressive chemotherapy treatments.

Though with encouraging outcomes, there are some limitations in this study. The first is the relatively small patient population, which may reduce the ability to detect minor potential statistically significant differences in clinical outcomes, AEs, and SAEs. Moreover, Trilaciclib showed its clinical efficacy in reducing the occurrence of CIM in treating SCLC in three clinical trials. Still, the metastatic triple-negative breast cancer trial observed no improvement in myelosuppression endpoints. Whether this is about gender differences, the type of cancer, or chemotherapy regimens needs to be determined. This underscores the need to explore this difference’s potential causes to confirm the clinical benefits of Trilaciclib further.

Together with these results, Trilaciclib demonstrated its efficacy in relieving patients from CIM-related side effects and improving the overall safety profile of myelosuppressive chemotherapy without inducing other unexpected side effects. These findings also support further clinical trials in a larger population and with more chemotherapy regimens in multiple types of cancers to demonstrate its clinical benefits.

## Data Availability

The original contributions presented in the study are included in the article/supplementary material, further inquiries can be directed to the corresponding authors.

## References

[B1] Bianchini G. De Angelis C. Licata L. Gianni L. (2022). Treatment landscape of triple-negative breast cancer - expanded options, evolving needs. Nat. Rev. Clin. Oncol. 19, 91–113. 10.1038/s41571-021-00565-2 34754128

[B2] Bohlius J. Bohlke K. Castelli R. Djulbegovic B. Lustberg M. B. Martino M. (2019). Management of cancer-associated anemia with erythropoiesis-stimulating agents: ASCO/ASH clinical practice guideline update. Blood Adv. 3, 1197–1210. 10.1182/bloodadvances.2018030387 30971397 PMC6482353

[B3] Crawford J. Denduluri N. Patt D. Jiao X. Morrow P. K. Garcia J. (2020). Relative dose intensity of first-line chemotherapy and overall survival in patients with advanced non-small-cell lung cancer. Support Care Cancer 28, 925–932. 10.1007/s00520-019-04875-1 31172284 PMC6954126

[B4] Deng J. Wang E. S. Jenkins R. W. Li S. Dries R. Yates K. (2018). CDK4/6 inhibition augments antitumor immunity by enhancing T-cell activation. Cancer Discov. 8, 216–233. 10.1158/2159-8290.CD-17-0915 29101163 PMC5809273

[B5] Dómine Gómez M. Csőszi T. Jaal J. Kudaba I. Nikolov K. Radosavljevic D. (2021). Exploratory composite endpoint demonstrates benefit of trilaciclib across multiple clinically meaningful components of myeloprotection in patients with small cell lung cancer. Int. J. Cancer 149, 1463–1472. 10.1002/ijc.33705 34109630 PMC8457063

[B6] Epstein R. S. Aapro M. S. Basu Roy U. K. Salimi T. Krenitsky J. Leone-Perkins M. L. (2020). Patient burden and real-world management of chemotherapy-induced myelosuppression: Results from an online survey of patients with solid tumors. Adv. Ther. 37, 3606–3618. 10.1007/s12325-020-01419-6 32642965 PMC7340862

[B7] Epstein R. S. Weerasinghe R. K. Parrish A. S. Krenitsky J. Sanborn R. E. Salimi T. (2022). Real-world burden of chemotherapy-induced myelosuppression in patients with small cell lung cancer: A retrospective analysis of electronic medical data from community cancer care providers. J. Med. Econ. 25, 108–118. 10.1080/13696998.2021.2020570 34927520

[B8] Ferrarotto R. Anderson I. Medgyasszay B. Garcia-Campelo M. R. Edenfield W. Feinstein T. M. (2021). Trilaciclib prior to chemotherapy reduces the usage of supportive care interventions for chemotherapy-induced myelosuppression in patients with small cell lung cancer: Pooled analysis of three randomized phase 2 trials. Cancer Med. 10, 5748–5756. 10.1002/cam4.4089 34405547 PMC8419768

[B9] Goldman J. W. Dvorkin M. Chen Y. Reinmuth N. Hotta K. Trukhin D. (2021). Durvalumab, with or without tremelimumab, plus platinum-etoposide versus platinum-etoposide alone in first-line treatment of extensive-stage small-cell lung cancer (CASPIAN): Updated results from a randomised, controlled, open-label, phase 3 trial. Lancet Oncol. 22, 51–65. 10.1016/s1470-2045(20)30539-8 33285097

[B10] Hart L. L. Ferrarotto R. Andric Z. G. Beck J. T. Subramanian J. Radosavljevic D. Z. (2021). Myelopreservation with trilaciclib in patients receiving topotecan for small cell lung cancer: Results from a randomized, double-blind, placebo-controlled phase II study. Adv. Ther. 38, 350–365. 10.1007/s12325-020-01538-0 33123968 PMC7854399

[B11] He S. Roberts P. J. Sorrentino J. A. Bisi J. E. Storrie-White H. Tiessen R. G. (2017). Transient CDK4/6 inhibition protects hematopoietic stem cells from chemotherapy-induced exhaustion. Sci. Transl. Med. 9, eaal3986. 10.1126/scitranslmed.aal3986 28446688 PMC5774632

[B12] Higgins J. P. T. Altman D. G. Gøtzsche P. C. Jüni P. Moher D. Oxman A. D. (2011). The Cochrane Collaboration’s tool for assessing risk of bias in randomised trials. BMJ 343, d5928. 10.1136/bmj.d5928 22008217 PMC3196245

[B13] Horn L. Mansfield A. S. Szczęsna A. Havel L. Krzakowski M. Hochmair M. J. (2018). First-Line atezolizumab plus chemotherapy in extensive-stage small-cell lung cancer. N. Engl. J. Med. 379, 2220–2229. 10.1056/NEJMoa1809064 30280641

[B14] Hussein M. Maglakelidze M. Richards D. A. Sabatini M. Gersten T. A. Lerro K. (2021). Myeloprotective effects of trilaciclib among patients with small cell lung cancer at increased risk of chemotherapy-induced myelosuppression: Pooled results from three phase 2, randomized, double-blind, placebo-controlled studies. Cancer Manag. Res. 13, 6207–6218. 10.2147/CMAR.S313045 34408488 PMC8363477

[B15] Kogan L. G. Davis S. L. Brooks G. A. (2019). Treatment delays during FOLFOX chemotherapy in patients with colorectal cancer: A multicenter retrospective analysis. J. Gastrointest. Oncol. 10, 841–846. 10.21037/jgo.2019.07.03 31602321 PMC6776798

[B16] Lai A. Y. Sorrentino J. A. Dragnev K. H. Weiss J. M. Owonikoko T. K. Rytlewski J. A. (2020). CDK4/6 inhibition enhances antitumor efficacy of chemotherapy and immune checkpoint inhibitor combinations in preclinical models and enhances T-cell activation in patients with SCLC receiving chemotherapy. J. Immunother. Cancer 8, e000847. 10.1136/jitc-2020-000847 33004541 PMC7534680

[B17] Powell K. Prasad V. (2021). Concerning FDA approval of trilaciclib (Cosela) in extensive-stage small-cell lung cancer. Transl. Oncol. 14, 101206. 10.1016/j.tranon.2021.101206 34419683 PMC8379686

[B18] Roberts P. J. Kumarasamy V. Witkiewicz A. K. Knudsen E. S. (2020). Chemotherapy and CDK4/6 inhibitors: Unexpected bedfellows. Mol. Cancer Ther. 19, 1575–1588. 10.1158/1535-7163.MCT-18-1161 32546660 PMC7473501

[B19] Tan A. R. Wright G. S. Thummala A. R. Danso M. A. Popovic L. Pluard T. J. (2019). Trilaciclib plus chemotherapy versus chemotherapy alone in patients with metastatic triple-negative breast cancer: A multicentre, randomised, open-label, phase 2 trial. Lancet Oncol. 20, 1587–1601. 10.1016/S1470-2045(19)30616-3 31575503

